# A Randomized, Double-Blind, Placebo and Active Controlled Phase II Study to Evaluate the Safety and Efficacy of Novel Dutasteride Topical Solution (0.01%, 0.02%, and 0.05% w/v) in Male Subjects With Androgenetic Alopecia

**DOI:** 10.7759/cureus.89309

**Published:** 2025-08-03

**Authors:** Veerendra Kumar Panuganti, Pavan Kumar Madala, Venkata Ramalingayya Grandhi, Chandrasekhar Varma Alluri, Javeed Mohammad, Sanyasi Rao KSSVV, Mamatha Reddy Dundigalla

**Affiliations:** 1 Clinical Affairs Department, Shilpa Medicare Limited, Nacharam Unit, Hyderabad, IND; 2 Bioanalytical Department, Shilpa Medicare Limited, Nacharam Unit, Hyderabad, IND; 3 Medical Affairs Department, Shilpa Medicare Limited, Nacharam Unit, Hyderabad, IND

**Keywords:** 5-alpha reductase selective inhibitors, androgenetic alopecia, dutasteride, finasteride, hair loss, male pattern alopecia

## Abstract

Introduction

Oral dutasteride has demonstrated superiority over finasteride in treating androgenetic alopecia (AGA). We have developed a novel topical dutasteride formulation, which has shown promising efficacy, safety, and tolerability in preclinical studies. The present study objective is to compare the efficacy and safety of dutasteride topical solutions (0.01%, 0.02%, and 0.05% w/v) with placebo and oral finasteride (1 mg/day) in AGA males.

Methods

In this phase II study, 135 AGA males (20-60 years of age) were randomized to receive dutasteride topical solution (0.01%, 0.02%, 0.05% w/v), finasteride (1 mg), or placebo daily for 24 weeks. The primary endpoint was target area hair count (TAHC) within 1 cm^2^ at the vertex at week 24. Secondary endpoints included TAHC at week 12 and target area hair width (TAHW), male hair growth questionnaire (MHGQ) score, and investigator global photography assessment (GPA) at week 12 and week 24.

Results

Dutasteride topical solution demonstrated a dose-dependent increase in TAHC and TAHW vs placebo at week 24 (p≤0.01). The 0.05% dutasteride solution significantly improved TAHC vs finasteride at week 24 (p=0.0083). More patients in the 0.05% dutasteride group achieved a GPA score of ≥+2 and an MHGQ score ≤ 2 at week 24 than those on finasteride. No irritation was reported in active treatment groups. Dutasteride caused modest changes in serum testosterone/dihydrotestosterone, while finasteride caused moderate changes.

Conclusion

Dutasteride topical solution (0.05% w/v) demonstrated to be more efficacious than finasteride (1 mg/day) in treating male AGA.

## Introduction

Male androgenetic alopecia (AGA), also known as “male pattern baldness,” is the most common form of baldness among males, affecting 58% of men aged 30-50 years in India and 50% of men worldwide [1]. AGA is characterized by the progressive hair loss of terminal hairs on the scalp in a characteristic distribution, typically involving the anterior, mid, temporal, and vertex sites. Dermoscopic signs of AGA include hair shaft thickness heterogeneity, follicles with a single hair, increased vellus hair, and perifollicular pigmentation [2]. Beyond its physical appearance, hair loss profoundly affects psychological well-being and quality of life, often leading to low self-esteem, depression, and social anxiety [3]. The pathogenesis of AGA involves the conversion of testosterone to dihydrotestosterone (DHT), a more potent androgen, facilitated by the enzyme 5-α-reductase (5AR). Elevated levels of DHT or increased sensitivity to DHT can result in miniaturization of the hair follicle, a shortened anagen phase of the hair cycle, and eventual hair loss [4]. Currently, only two pharmacological treatments have been approved by the United States Food and Drug Administration (FDA) for AGA: topical minoxidil and oral finasteride (1 mg/day).

The 5-alpha reductase selective inhibitors (5ARIs), viz., finasteride and dutasteride, were initially introduced as therapeutic agents for the treatment of benign prostatic hyperplasia in 1992 and 2002, respectively [5]. The introduction of 5ARIs for the treatment of AGA has significantly transformed the therapeutic landscape. Both finasteride and dutasteride are effective in reducing male pattern hair loss by lowering levels of DHT. Finasteride, a type-II 5ARI, has been demonstrated to significantly improve hair growth [6], delay hair loss as compared to a placebo [7-9], and is frequently used in AGA treatment. However, 30-50% of patients failed to demonstrate clinical improvement following finasteride treatment [10-12]. Although finasteride is efficacious for hair regrowth, its systemic use is associated with significant side effects, limiting long-term utilization. The treatment of AGA should aim not only to arrest AGA progression, but also to increase hair density and thickness, thereby improving the quality of life [13]. This underscores the need to search for alternative treatment modalities with better efficacy and safety.

Recently, dutasteride, a dual 5ARI (selectively inhibits both type-I and II 2, 5AR isoenzymes), has emerged as a potential treatment for AGA with off-label use. Several randomized clinical trials have provided evidence of oral dutasteride’s efficacy in mild to moderate AGA and have demonstrated its superiority over placebo and finasteride as well [14-18]. As reported, dutasteride is approximately three times more potent than finasteride in inhibiting type-II 5AR and 100 times more potent in inhibiting type-I 5AR [19]. Dutasteride may serve as an alternative treatment option for AGA due to the inconspicuous efficacy of finasteride. The oral formulation of dutasteride is associated with less but elevated incidence of side effects such as sexual dysfunction, decreased libido, and in some cases, depression [14-16]. However, the topical formulation would have minimal systemic absorption, resulting in minimal systemic side effects and better drug availability at the site of action, i.e., skin of the scalp, thereby offering better efficacy without any safety concerns.

A novel topical solution of dutasteride is a viable option for individuals at risk for, experiencing, or concerned about these side effects. This controlled Phase II clinical trial reports the efficacy and safety of dutasteride topical solutions (0.01%, 0.02%, and 0.05% w/v), as compared to an oral placebo and finasteride oral tablets (1 mg) in male participants with AGA (primary endpoint). This study further compared serum testosterone and DHT levels among the treatment arms, as well as assessed the pharmacokinetic profile of 0.05% dutasteride topical solution (secondary endpoints).

## Materials and methods

This was a phase II, multi-centre, randomized, double-blind, double-dummy, parallel-group, active- and placebo-controlled study conducted across the following six centers in India: RajaRajeswari Medical College & Hospital (Bengaluru), Lifepoint Research (Pune), Anand Multispecialty Hospital (Vadodara), Osmania Medical College (Hyderabad), Sai Sneh Hospital and Diagnostic Centre (Pune), and Kempegowda Institute of Medical Sciences (Bangalore). It was approved by the institutional ethics committees of these centers. The trial was registered at the Clinical Trial Registry-India (CTRI) with the registration number CTRI/2021/03/032173 dated March 19, 2021, and reported according to the Consolidated Standards of Reporting Trials (CONSORT) 2025 guidelines.

The trial was conducted according to Good Clinical Practice (GCP) guidelines developed by the International Council for Harmonisation, Indian GCP Guidelines, New Drugs and Clinical Trials Rules 2019 (G.S.R. 227(E)), Indian Council of Medical Research, National Ethical Guidelines for Biomedical and Health Research involving Human Participants, and the principles enunciated in the Declaration of Helsinki (World Medical Association General Assembly, Fortaleza, Brazil, October 2013). All participants provided written informed consent prior to their enrollment in the study. For a Phase II study, a formal sample size estimation is generally not required; therefore, it was not computed.

Eligibility criteria

Inclusion criteria were individuals of the male sex, aged 20-60 years, with types III vertex, IV and V male pattern hair loss (AGA) according to the Norwood-Hamilton classification criteria [20], who had not received treatment with dutasteride or finasteride in the past 12 months, and who agreed to mark the target area and maintain the same hair length throughout the study period. Patients with a history or evidence of hair loss other than AGA (e.g., due to autoimmune, endocrine, mechanical, or infectious processes or secondary to a scalp dermatological disorder), those who underwent hair transplant surgery or hair weaving, light and laser treatment on the scalp, or exhibited skin damage such as skin abrasions, actinic keratosis, or any abnormal findings on the scalp were excluded. Other main exclusion criteria included suspicion of malignancy, including prostate cancer, a history of active seborrheic dermatitis, varicocele, or hypersensitivity, a history of infertility or difficulty in fathering children, or a history of active unstable thyroid disease. Individuals who made concurrent use of systemic corticosteroids, topical corticosteroids, anabolic steroids, or over-the-counter medications (e.g., minoxidil) were also excluded. Use of drugs with anti-androgenic properties within six months of study entry (including flutamide, cyproterone acetate, estrogen, progesterone, cimetidine, spironolactone, or ketoconazole) or use of any of medications such as strong inhibitors of CYP3A4, strong inducers of CYP3A4, drugs associated with QT prolongation, antihypertensive, and other drugs known to cause hypotension within 14 days prior to enrolment, were also part of exclusion criteria for the study.

Randomization and masking

The randomization schedule was generated using PROC PLAN (SEED=) of SAS® Studio 3.6 (SAS Institute Inc., Cary, North Carolina, United States). The randomization scheme and kit numbers were provided by a third-party organisation (Bilcare Research, Caprihans India Limited, Pune, India). Patients were assigned randomly to five treatment groups in a 2:2:2:2:1 ratio: (i) Dutasteride 0.01% w/v topical solution (T1) + Oral finasteride placebo (P2) (n=30), (ii) Dutasteride 0.02% w/v topical solution (T2) + Oral finasteride placebo (P2) (n=30), (iii) Dutasteride 0.05% w/v topical solution (T3) + Oral finasteride placebo (P2) (n=30) (iv) Dutasteride placebo topical solution (P1) + Oral finasteride 1 mg tablets (R), (n=30), and (v) Dutasteride placebo topical solution (P1) + Oral finasteride placebo (P2) (n=15). Each patient was assigned a unique four-digit randomization number (e.g., R001, R002) across all study sites, which appeared in individual listings and figures of the clinical study and its report.

All study products were identically packaged to maintain blinding. Treatment assignments remained blinded to patients, investigators/study staff, and the sponsor/contract research organization (CRO) overseeing the trial. The IWRS or appropriate method was utilized for treatment allocation throughout the study.

Composition of dutasteride topical solution

The composition of the dutasteride topical solution is given in Table 1.

**Table 1:** Composition of Dutasteride Topical Solution

Ingredient	Grade	Function	Dutasteride, 0.01%, 0.1 mg/ml topical solution		Dutasteride, 0.02%, 0.2 mg/ml topical solution		Dutasteride, 0.05%, 0.5 mg/ml topical solution	
									Quantity					
			mg/g	% w/w	mg/g	% w/w	mg/g	% w/w
			Dutasteride	USP/Ph.Eur	Active ingredient	0.11	0.01	0.22	0.02	0.55	0.05
Dehydrated alcohol	USP/Ph.Eur	Penetration enhancer	299.89	29.99	299.78	29.98	299.44	29.94
Medium-chain triglycerides	NF/Ph.Eur	Solubilizer	300	30	300	30	300	30
									Castor oil	USP/Ph.Eur	Viscosity enhancer	400	40	400	40	400	40

Interventions and drug administration

For test participants (Tl/T2/T3), 1 ml of dutasteride topical solution (0.01%, 0.02%, 0.05% w/v, respectively) was applied with a dropper directly over a targeted 1.9 cm^2^ circular area of hair loss on the scalp once daily for 24 weeks, followed by scalp massage with fingers [21]. The solution was left in place for at least six to eight hours. Dutasteride topical solutions, 0.01%, 0.02%, and 0.05% w/v contain 0.1 mg, 0.2 mg, and 0.5 mg of dutasteride per ml, respectively. They were also administered oral finasteride placebo (P2), once daily for 24 weeks.

For reference (R) participants, 1 mg oral tablets of finasteride were administered once daily in the morning for 24 weeks, and 1 ml of dutasteride placebo (P1) topical solution was applied with a dropper directly onto the scalp in the hair loss area once daily for 24 weeks, followed by scalp massage with fingers. 

The total study duration was 28 weeks, which included a two-week screening phase, treatment up to week 24, and follow-up at week 28. Treatment compliance was evaluated by maintaining a daily diary and drug accountability log. Only patients with compliance within the range of 80-120%, based on the number of days treated, were allowed to continue in the study.

Study endpoints

The primary efficacy endpoint was the change from baseline in the target area hair count (TAHC) (density) within 1 cm^2^ at the vertex at week 24, as assessed by macrophotography. The secondary efficacy endpoints were: (a) change from baseline in the TAHC (density) within 1 cm^2^ at the vertex at week 12, as assessed by macrophotography, (b) change from baseline in the target area hair width (TAHW) (thickness) within 1 cm^2^ at the vertex at week 12 and week 24, as assessed by macrophotography, (c) the self-administered male hair growth questionnaire (MHGQ) (see Appendices) score at week 12 and week 24, (d) investigator global photography assessment (GPA) score of TAHC and TAHW from baseline to week 12 and week 24.

Efficacy assessments

Selection of Treatment Site

Following randomization, the target treatment site was selected as follows: (i) The patient's hair was combed away from the vertex bald spot to make the entire balding area visible. (ii) A circular area of approximately 1.9 cm^2^ was identified in the anterior leading edge of the vertex thinning area. The area was clipped to approximately 0.5 mm to 1 mm in length and marked appropriately in the center of the circle of the clipped hair at a 90° angle. (iii) After the hair was clipped, the area was cleaned with a stick tape to remove clipped hair stubble. (iv) A global photograph of the patient’s vertex scalp was taken.

Assessments

Investigator global photography assessment: Digital color photographs of the patient's vertex scalp were taken of the head after the hair was combed away from the vertex bald spot to make the entire balding area visible at baseline (day 1), week 12, and week 24 or at the early termination visit. Investigators assessed changes in hair growth from baseline to week 12 and week 24 in TAHC and TAHW at the vertex using a 7-point scale: -3=greatly decreased, -2=moderately decreased, -1=slightly decreased, 0=no change, +1=slightly increased, +2=moderately increased, and +3=greatly increased [22].

Macrophotography: Macrophotography of the scalp was conducted using a Dino-Lite Digital Microscope camera (Absolute Data Services Ltd, Hemel Hempstead, United Kingdom). The camera was set to specific magnifications for different parameters: growth rate (60x), thickness (200x), density (60x), and condition of scalp (60x). Bright light-emitting diode (LED) lighting was employed to ensure optimal illumination of the scalp during image capture. The microscope was positioned to touch the surface of the scalp during image acquisition. Hair counts along with TAHW were measured at baseline (day 1), week 12, and week 24, or at the early termination visit.

MHGQ: Patients assessed their scalp hair using a self-administered MHGQ, consisting of four questions in the patient’s language on treatment efficacy and three questions on satisfaction with appearance. The questionnaire was administered to eligible participants to subjectively measure their perception of hair growth at week 12 and week 24. The self-administered MHGQ overall score was assessed using the following five-point scale: 1=very satisfied, 2=satisfied, 3=neutral (neither satisfied nor dissatisfied), 4=dissatisfied, 5=very dissatisfied. A higher score indicated a worse outcome [23]. The lower the MHGQ score, the higher the improvement in patient satisfaction and thereby better hair growth.

Safety assessments

Skin Irritation

Local tolerability at the application sites was assessed at the clinic one hour post-dose at baseline (day 1), week 4, week 8, week 12, and week 24, or at the early termination visit. The severity score for skin irritation was recorded using a dermal response scale ranging from 1 to 8: a score of 1 indicates no evidence of irritation; 2, minimal, barely perceptible erythema; 3, definite erythema that was readily visible, with minimal oedema or minimal papular response; 4, erythema and papules; 5, definite oedema; 6, erythema, oedema, and papules; 7, vesicular eruption; and 8, strong reaction spreading beyond test site. Additional local effects were categorized alphabetically, where A indicates a slight glazed appearance; B, marked glazing; C, glazing with peeling and cracking; D, glazing with fissures; E, film of dried serous exudate covering part or all of the patch site; and F, small petechial erosions or scabs.

Adverse Events

The incidence of drug-related adverse events, as assessed by clinical examinations, vital signs monitoring, and/or laboratory results.

Laboratory Tests

Blood and urine samples were collected at the screening visit (baseline) and at week 24 or early termination visit to perform safety laboratory tests.

DHT and Testosterone

DHT and testosterone levels were assessed prior to drug administration at baseline (day 1), week 4, week 8, week 12, and week 24 or early termination visit.

Pharmacokinetic assessments

Blood samples for pharmacokinetic analysis were collected on day 1 (pre-dose (0.00), up to 24 hours post-dose (0.25, 0.50, 1.00, 2.00, 3.00, 4.00, 5.00, 6.00, 8.00, 12.00, and 24.00 (day 2)) and on day 168 (Week 24). Blood samples were collected in potassium ethylenediaminetetraacetic acid (K2EDTA) through an indwelling intravenous cannula placed in a forearm vein. Samples were centrifuged at 3000 revolutions per minute (RPM) at 4°C for 10 minutes. The plasma was transferred into two pre-labelled polypropylene sample tubes as two aliquots (2.0 ml of plasma was transferred to aliquot 1, and the remaining plasma was transferred to aliquot 2). The aliquots were placed in an ice bath for 15 minutes. Both aliquot vials were stored upright in a box containing dry ice or in a freezer at a temperature of -20°C ± 10°C until the shipment reached the final destination for bioanalysis. All pharmacokinetic samples were analyzed to measure dutasteride concentration using a validated liquid chromatography tandem mass spectrometry (LC-MS/MS) method developed at Shilpa Medicare Limited, India. The method employed solid phase extraction for sample preparation and utilized dutasteride-13C6 as the internal standard. The assay was validated over a concentration range of 50.125 to 10024.931 pg/ml, with a lower limit of quantification (LLOQ) of 50.125 pg/ml.

Statistical analysis

Statistical analysis was performed using SAS® Studio 3.6 (SAS Institute Inc.). Statistical analysis was conducted on data from all patients who had measurements at both baseline and during treatment (intention-to-treat population). Safety assessments included all randomized patients who received at least one application of the investigational drug. Continuous data were reported as mean (± SD), range, while categorical data were reported as number (percentage).

To compute the mean change from baseline in variables, analysis of covariance (ANCOVA) employing a mixed linear model using the PROC MIXED procedure in the SAS software was used. This model included treatment, center, visit, and treatment-by-visit interaction as fixed effects, with baseline as a covariate. Since the primary endpoint is the change from baseline in TAHC, baseline is taken as a covariate, and a significant change from baseline in TAHC was observed in the study.

The variance-covariance matrix of unstructured form was used to model correlations between two repeated measurements (over the post-baseline visits) within each patient. A two-sided test with a p-value ≤ 0.05 was considered statistically significant.

Pharmacokinetic parameters were estimated using Phoenix WinNonlin™ version 8.3 (Certara Inc., Radnor, Pennsylvania, United States). Missing samples (M) and non-reportable values (NRV) were considered as missing, and the concentration values below the limit of quantification (BLQ) were considered as zero. Since the Dutasteride product is intended for topical application, the systemic exposures are expected to be very low, and are near or below the lower limit of quantification (LLOQ) even at the highest dose applied; hence, only highest dosage strength group (i.e., Dutasteride 0.05% w/v (0.5 mg/ml)) was considered for pharmacokinetic assessment. Patients meeting the following criteria were excluded from the pharmacokinetic analysis: (i) patients with pre-dose concentration > 5% of the C_max_, (ii) patients with less than three quantifiable post dose concentrations, or (iii) patients excluded from the analysis of AUC_0-inf_, t_1/2_, K_el_, if they did not have at least three measurable concentrations after C_max_.

## Results

**Figure 1:**
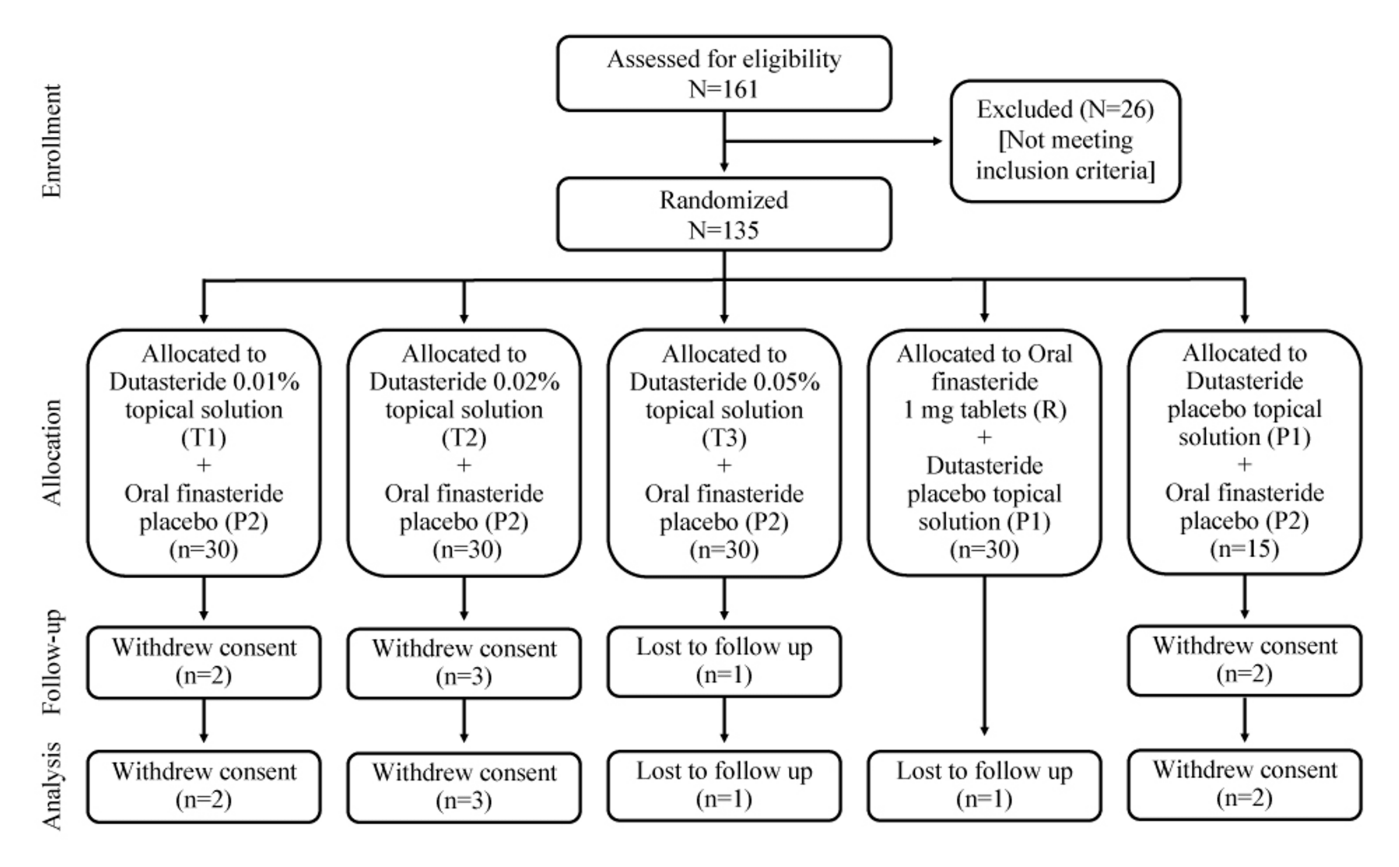
CONSORT flow diagram CONSORT: Consolidated Standards of Reporting Trials

A total of 161 AGA patients were screened, and the randomization of 135 patients from six centers began on April 14, 2021, and ended on May 5, 2022. Of the total randomized patients, 131 completed week 4 and week 8 visits, and 128 patients completed the week 12 visit of the study. In total, 127 patients completed the entire study (up to week 24) and were included in the primary and secondary endpoint analysis. Pharmacokinetic sample concentration data for dutasteride were collected from 16 patients of the dutasteride 0.05% cohort. A CONSORT flow diagram illustrating the progress of participants through the phases of the trial is illustrated in Figure 1.

Patient’s demographics and baseline characteristics were similar across the five treatment groups (Table 2). The mean age of the study population was 38.1 ± 9.4 years (range: 20-58 years). All patients with AGA were male and Asian.

**Table 2:** Baseline demographic characteristics Data are reported as mean± SD, n, and n (%) as marked

Variables	Dutasteride 0.01% topical solution (n=30)	Dutasteride 0.02% topical solution (n=30)	Dutasteride 0.05% topical solution (n=30)	Oral finasteride 1 mg tablets (n=30)	Placebo (n=15)
Age (years), mean ± SD (range)	37.80 ± 10.10 (21–53)	41.20 ± 9.60 (25–58)	37.10 ± 8.20 (20–54)	37.20 ± 9.30 (22–56)	36.30 ± 10.00 (23–54)
Age group (years), n (%)					
18–40	20 (66.67)	16 (53.33)	23 (76.67)	23 (76.67)	11 (73.33)
41–64	10 (33.33)	14 (46.67)	7 (23.33)	7 (23.33)	4 (26.67)
Sex (male), n	30	30	30	30	15
Ethnicity					
Non-Hispanic or Latino	30	30	30	30	15
Asian	30	30	30	30	15
Height, mean ± SD (range)	168.70 ± 9.40 (152–201.1)	167.50 ± 7.20 (155–180)	166.60 ± 8.30 (151–188.9)	167.30 ± 6.40 (155–179)	165.20 ± 7.40 (150–180)
Weight, mean ± SD (range)	69.41 ± 9.63 (47–95.40)	68.36 ± 10.65 (49–98.5)	67.25 ± 10.16 (52–95.70)	70.88 ± 10.90 (51–102.1)	70.40 ± 10.15 (54.5–96.4)
Norwood-Hamilton stage, n (%)					
Stage III vertex	11 (36.67)	7 (23.33)	12 (40)	7 (23.33)	6 (40)
Stage IV	11 (36.67)	14 (46.67)	9 (30)	16 (53.33)	6 (40)
Stage V	8 (26.67)	9 (30)	9 (30)	7 (56.67)	3 (20)
Chronic smoker, n (%)	0 (0.00	0 (0.00)	0 (0.00)	0 (0.00)	0 (0.00)
Alcohol consumption (>2 drinks or equivalent per day), n (%)	0 (0.00)	0 (0.00)	0 (0.00)	0 (0.00)	0 (0.00)
Medical history, n (%)	0 (0.00)	0 (0.00)	0 (0.00)	0 (0.00)	0 (0.00)
History of past treatment, n (%)	0 (0.00)	0 (0.00)	0 (0.00)	0 (0.00)	0 (0.00)
Baseline hair count (per cm^2^), mean ± SD	318.65 ± 93.52	322.87 ± 87.49	327.93 ± 91.84	305.73 ± 79.75	316.78 ± 88.36
Baseline hair width (µm), mean ± SD	28.10 ± 9.17	26.9 ± 8.96	30.38 ± 10.03	28.73 ± 10.62	29.51 ± 9.96

Change from baseline in the TAHC at week 24 and week 12****

All active treatment groups demonstrated substantial improvement in TAHC within 1 cm² at the vertex at week 24 compared to baseline (all p ≤ 0.01). Dutasteride significantly increased TAHC than placebo in a dose-dependent manner at week 24 (p ≤ 0.01). Dutasteride topical solution (0.01% and 0.05%) and finasteride showed an increase in TAHC significantly better than placebo at week 24 (all p < 0.05). Pairwise comparisons revealed a significant improvement in TAHC with 0.05% dutasteride compared to finasteride at week 24 (p=0.0083) (primary efficacy endpoint). There was a statistically significant improvement in TAHC with dutasteride 0.05% topical solution than placebo at week 12 (p=0.038) (secondary efficacy endpoint)(Table 3).

**Table 3:** Mean change from baseline in target area hair count (TAHC) at week 24 and week 12 * P≤ 0.05 and ^@^P≤ 0.01 were considered statistically significant. SEM: standard error of mean

Treatment Group	Baseline to Week 24				Baseline to Week 12			
	Mean difference	SEM	t-value	P-value	Mean difference	SEM	t-value	P-value
Least squares mean changes from baseline								
Dutasteride 0.01% topical solution (n=28)	32.32	9.12	3.55	0.0006^@^	10.79	9.04	1.19	0.23
Dutasteride 0.02% topical solution (n=27)	27.48	9.28	2.96	0.0037^@^	-1.15	9.21	-0.12	0.90
Dutasteride 0.05% topical solution (n=29)	75.52	8.96	8.43	0.0001^@^	31.53	8.74	3.61	0.0004^@^
Oral finasteride 1 mg tablets (n=29)	41.21	9.12	4.52	0.0001^@^	11.50	9.04	1.27	0.20
Placebo (n=15)	0.07	12.46	0.01	0.9957	-0.27	12.36	-0.02	0.98
Pair-wise comparisons between treatments								
Placebo vs Dutasteride 0.01% topical solution	-32.25	15.44	-2.09	0.0388*	-11.05	15.31	-0.72	0.47
Placebo vs Dutasteride 0.02% topical solution	-27.41	15.53	-1.76	0.0801	0.88	15.41	0.06	0.95
Placebo vs Dutasteride 0.05% topical solution	-75.45	15.34	-4.92	0.0001^@^	-31.80	15.13	-2.10	0.038*
Placebo vs Oral finasteride 1 mg tablets	-41.15	15.44	-2.67	0.0087^@^	-11.77	15.31	-0.77	0.44
Dutasteride 0.05% topical solution vs Oral finasteride 1 mg tablets	34.30	12.78	2.68	0.0083^@^	20.03	12.57	1.59	0.11

Change from baseline in the TAHW at week 12 and week 24

Dutasteride significantly increased TAHW as compared to placebo in a dose-dependent manner at week 24 (p ≤ 0.05). Dutasteride and finasteride significantly improved TAHW at week 24 relative to the baseline (p ≤ 0.05). Dutasteride 0.05% topical solution (p=0.0185) and finasteride (p=0.0387) showed significant improvement in efficacy, increasing TAHW relative to placebo at week 24 (Table 4).

**Table 4:** Mean change from baseline in target area hair width (TAHW) at week 24 and week 12 *P≤ 0.05 and ^@^P≤ 0.01 were considered statistically significant. SEM: standard error of mean

Treatment Group	Baseline to Week 24				Baseline to Week 12			
	Mean difference	SEM	t-value	P-value	Mean difference	SEM	t-value	P-value
Least squares mean changes from baseline								
Dutasteride 0.01% topical solution (n=28)	6.68	1.89	3.54	0.0006^@^	3.89	1.60	2.43	0.0164*
Dutasteride 0.02% topical solution (n=27)	9.15	1.92	4.76	0.0001^@^	5.44	1.63	3.34	0.0011^@^
Dutasteride 0.05% topical solution (n=29)	11.59	1.85	6.25	0.0001^@^	4.80	1.54	3.11	0.0023^@^
Oral finasteride 1 mg tablets (n=29)	10.68	1.89	5.66	0.0001^@^	6.35	1.60	3.98	0.0001^@^
Placebo (n=15)	4.00	2.58	1.55	0.1235	2.73	2.18	1.25	0.2133
Pairwise comparisons between treatments								
Placebo vs Dutasteride 0.01% topical solution	-2.68	3.19	-0.84	0.4036	-1.1595	2.71	-0.43	0.67
Placebo vs Dutasteride 0.02% topical solution	-5.15	3.22	-1.60	0.1120	-2.7111	2.72	-0.99	0.32
Placebo vs Dutasteride 0.05% topical solution	-7.59	3.18	-2.3	0.0185*	-2.0667	2.67	-0.77	0.44
Placebo vs Oral finasteride 1 mg tablets	-6.68	3.19	-2.09	0.0387*	-3.6238	2.71	-1.34	0.18
Dutasteride 0.05% topical solution vs Oral finasteride 1 mg tablets	0.91	2.65	0.34	0.7322	-1.5571	2.22	-0.70	0.48

Investigator global photography assessment score

**Figure 2:**
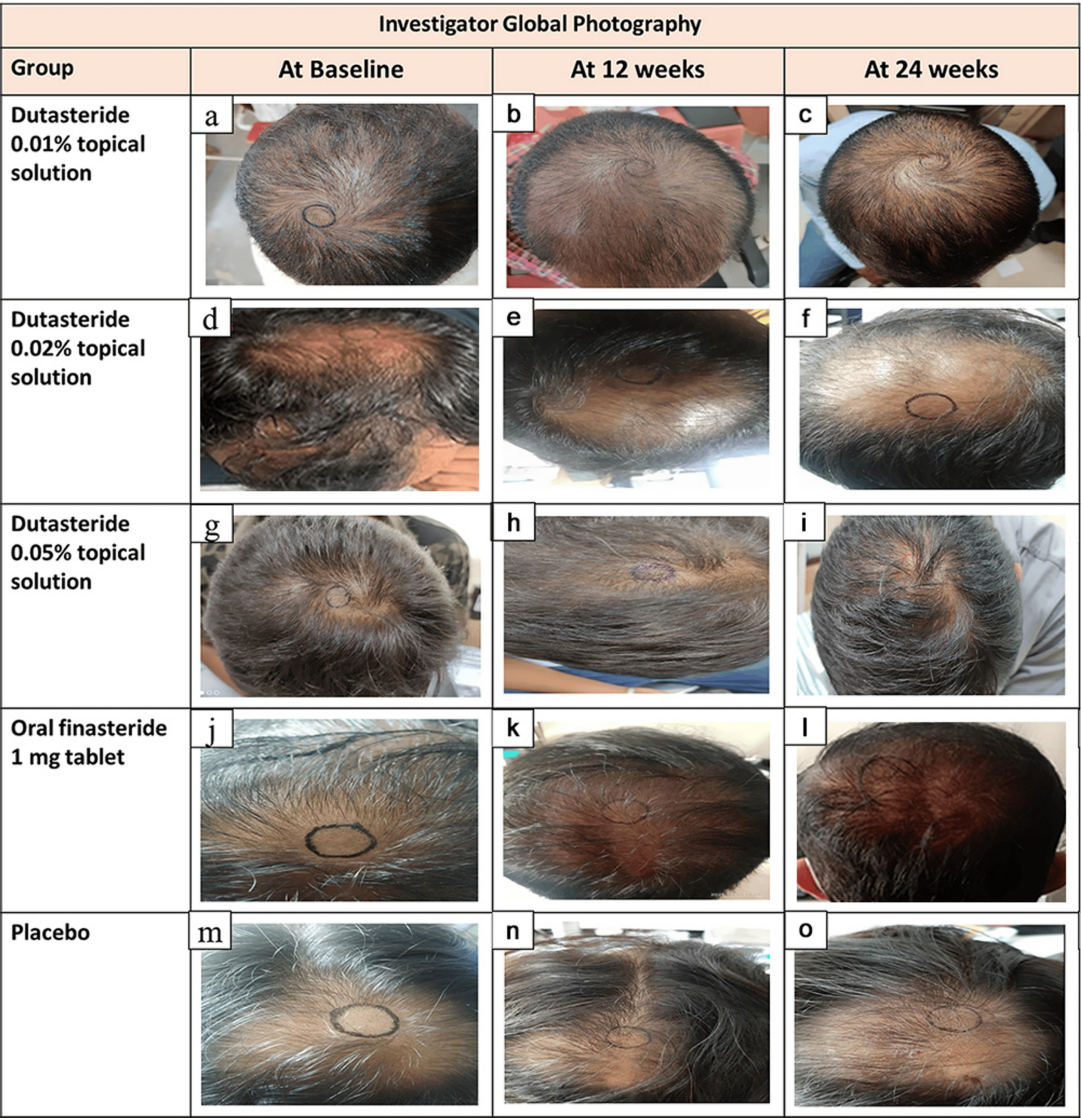
Representative images of hair growth in male-pattern androgenetic alopecia after treatment with 0.01% dutasteride topical solution at 12 week (b) and 24 week (c) vs baseline (a); 0.02% dutasteride topical solution at 12 week (e) and 24 week (f) vs baseline (d); 0.05% dutasteride topical solution at 12 week (h) and 24 week (i) vs baseline (g); oral finasteride 1 mg tablets at week 12 (k) and week 24 (l) vs baseline (j); placebo at week 12 (n) and week 24 (o) vs baseline (m).

As demonstrated in Table 5, the percentage of AGA patients with an investigator GPA score ≥ 2 for the hairline on top of their head was found to be high with dutasteride topical solution (0.05%), which is more than the reference finasteride cohort and placebo treatment. This indicates that a greater number of patients experienced enhanced hair growth and great improvement on investigator global photography on treatment with the dutasteride, 0.05% topical solution treatment when compared to other treatments at week 12 and week 24 (secondary efficacy endpoint). Improvements in hair growth at weeks 12 and 24 were compared to the baseline for all study groups and are evident in the investigator’s global photographs as representative images provided in Figure 2.

**Table 5:** AGA patients with investigator GPA score ≥ 2 at week 12 and week 24 *total number of patients was 29 in this category at week 24 AGA: androgenetic alopecia; GPA: global photography assessment

Treatment Group	Week 12		Week 24	
	Number of Patients With Score ≥ 2	Percentage	Number of Patients With Score ≥ 2	Percentage
Dutasteride 0.01% topical solution (n=28)	4	14.29	6	21.43
Dutasteride 0.02% topical solution (n=27)	10	37.03	14	51.85
Dutasteride 0.05% topical solution (n=30)	15	50	20*	68.97
Oral finasteride 1 mg tablets (n=28)	7	25	6	21.43
Placebo (n=15)	2	13.33	2	13.33

MHGQ score

As shown in Table 6, the percentage of AGA patients with a score ≤ 2 for hairline at the front of the head, the hairline on the top of the head, and overall hair appearance was found to be higher with dutasteride topical solution (0.05%) compared to the reference finasteride cohort and placebo treatment. This indicates that a greater number of patients were highly satisfied with the 0.05% dutasteride topical solution treatment compared to other treatments at both week 12 and week 24 (secondary efficacy endpoint)

**Table 6:** AGA patients with MHGQ score ≤ 2 for the hairline at the front of the head, the top of the head, and overall hair assessment at week 12 and 24 AGA: androgenetic alopecia; MHGQ: Male Hair Growth Questionnaire

Group	Week 12		Group	Week 24	
	Number of Patients With Score ≤ 2	Percentage		Number of Patients With Score ≤ 2	Percentage
The Hairline at the Front of Your Head?					
Dutasteride 0.01% topical solution (n=28)	20	71.43	Dutasteride 0.01% topical solution (n=28)	22	78.57
Dutasteride 0.02% topical solution (n=27)	18	66.67	Dutasteride 0.02% topical solution (n=27)	25	92.59
Dutasteride 0.05% topical solution (n=30)	28	93.33	Dutasteride 0.05% topical solution (n=29)	27	93.1
Oral finasteride 1 mg tablets (n=28)	18	64.29	Oral finasteride 1 mg tablets (n=28)	20	71.42
Placebo (n=15)	9	60	Placebo (n=15)	7	46.67
The Hair on Top of Your Head?					
Dutasteride 0.01% topical solution (n=28)	21	75	Dutasteride 0.01% topical solution (n=28)	20	71.43
Dutasteride 0.02% topical solution (n=27)	19	70.37	Dutasteride 0.02% topical solution (n=27)	24	88.89
Dutasteride 0.05% topical solution (n=30)	24	80	Dutasteride 0.05% topical solution (n=29)	28	96.55
Oral finasteride 1 mg tablets (n=28)	14	50	Oral finasteride 1 mg tablets (n=28)	20	71.43
Placebo (n=15)	8	53.33	Placebo (n=15)	5	33.33
Your Hair Overall?					
Dutasteride 0.01% topical solution (n=28)	24	85.71	Dutasteride 0.01% topical solution (n=28)	23	82.14
Dutasteride 0.02% topical solution (n=27)	19	70.37	Dutasteride 0.02% topical solution (n=27)	27	100
Dutasteride 0.05% topical solution (n=30)	26	86.67	Dutasteride 0.05% topical solution (n=29)	27	93.1
Oral finasteride 1 mg tablets (n=28)	18	64.29	Oral finasteride 1 mg tablets (n=28)	20	71.43
Placebo (n=15)	7	46.67	Placebo (n=15)	7	46.67

Safety evaluation

No adverse events, serious adverse events, deaths, or withdrawals of participation due to safety concerns were reported during the entire study period. Up to 28 weeks of follow-up, most of the patients (n=29; 16.96%) in active treatment groups scored 1 (indicated “no evidence of irritation”) in dermal assessments. Additionally, “slightly glazed appearance”, “Marked glazing”, and “Glazing with peeling and cracking” were observed across all treatment groups. There was no evidence of clinically significant laboratory abnormalities in the post-study evaluations.

**Figure 3:**
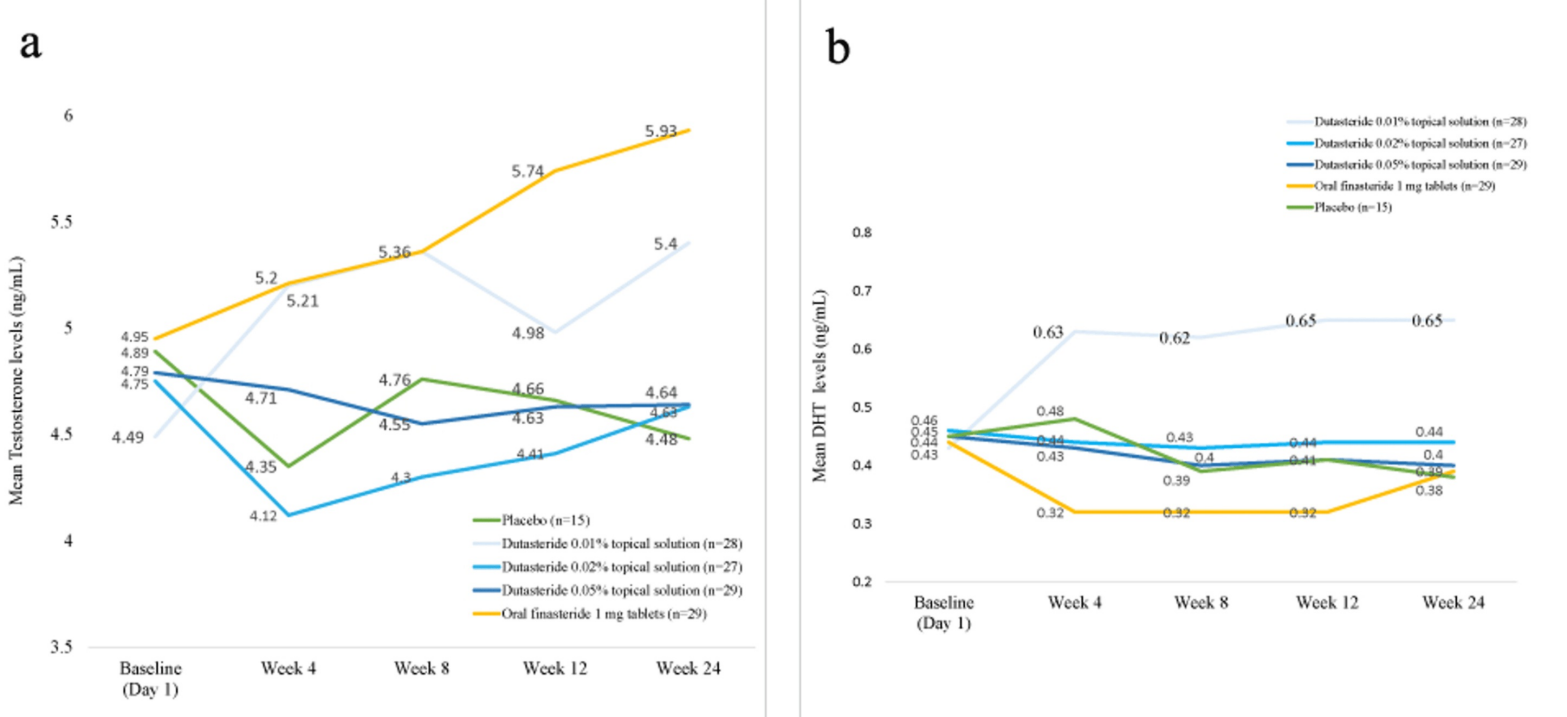
Changes from baseline to week 24 in (a) testosterone levels and (b) DHT levels DHT: dihydrotestosterone

In the oral finasteride group, testosterone levels increased by 16% at week 12 and continued to rise, reaching a 20% increase by week 24. Conversely, the group treated with topical dutasteride 0.05% solution showed modest changes at both week 12 and week 24. For the oral finasteride group, DHT levels decreased by 27% at week 12 but showed an incremental increase to an 11% reduction by week 24. In the topical dutasteride group, DHT levels decreased modestly by 8.9% at week 12 and maintained at an 11% reduction by week 24. No significant change from baseline in either serum testosterone or DHT concentrations was observed with dutasteride-treated groups at week 12 and week 24, whereas a moderate increase in testosterone and a moderate reduction in DHT (although not significant) was observed with the reference finasteride-treated group. Modest changes in testosterone and DHT were observed with 0.05% topical dutasteride solution (Figure 3).

Pharmacokinetic assessment

The mean time to attainment of C_max_ was approximately five hours for topical dutasteride solution (0.05% w/v) following a single-dose administration (Table 7). Following the peak, plasma concentrations declined slowly, with the initial elimination rate ranging from 11 to 13 hours. Most plasma concentrations were around or below the lower limit of quantification (LLOQ) up to 24 hours and even up to day 168. AUC_0-t_, AUC_0-inf_, and K_el _were not calculated due to very low exposure noticed in the pharmacokinetic population. The minimum and maximum exposure observed during the first 24 hours were 0 and 169.47 pg/ml, and the same values for day 168 were 0 and 150.51 pg/ml.

**Table 7:** Mean plasma concentration-time profile of 0.05% dutasteride topical solution following single dose administration up to 24 hours and then after multiple daily dose administrations on day 168 (n = 16). Exposures were found to be below or around the lower limit of quantification.

Pharmacokinetic sample time points (hours)	0.00	0.25	0.50	1.00	2.00	3.00	4.00	5.00	6.00	8.00	12.00	24.00	Day 168
Mean concentration (pg/ml)	0.00	4.0248	5.5802	17.826	6.8883	14.546	8.1173	169.47	31.609	11.096	3.6558	0.0000	15.904
Minimum concentration (pg/ml)	0.00	0.0000	0.0000	0.0000	0.0000	0.0000	0.0000	0.0000	0.0000	0.0000	0.0000	0.0000	0.0000
Maximum concentration (pg/ml)	0.00	64.397	89.283	115.30	55.647	182.47	73.546	2555.2	313.33	177.54	58.492	0.0000	150.51
SD	0.00	16.099	22.321	38.855	18.823	46.502	22.402	636.79	89.149	44.386	14.623	0.0000	44.281
CV%	---	400.00	400.00	217.97	273.27	319.69	275.98	375.77	282.04	400.00	400.00	---	278.43

## Discussion

The preclinical studies conducted on the test product, dutasteride topical solution, earlier had revealed promising efficacy, tolerability, and safety profiles (internal data; not published), supporting the initiation of a Phase II study. The acute dermal lethal dose (LD50) of dutasteride topical solution 1.0% w/v has been found to be > 2000 mg/kg in female Wistar rats. The no observed adverse effect level (NOAEL) for dutasteride topical solution in male rats has been determined to be < 0.1% w/v when applied at 500 mg/kg body weight/day in male rats. Dutasteride topical solution 1.0% w/v is classified as “Non-Irritant” to the eyes of New Zealand White rabbits and Guinea pigs (internal data; not published). Dutasteride topical solution was tolerated in 14-day (at 0.1%, 0.3%, and 1.0% w/v when applied dermally at 2000 mg/kg under semi-occlusive conditions for six hours for 14 days) and 90-day repeated dose dermal toxicity studies (0.1%, 0.3%, and 1.0% w/v when applied dermally at 500 mg/kg under semi-occlusive conditions for six hours for 90 consecutive days) in Sprague-Dawley male rats as well as 90-day repeated dose toxicity in New Zealand White male Rabbits (0.1, 0.3, and 1.0% w/v) (internal data; not published). In the current study, we compared the safety and efficacy of a topical dutasteride formulation with an oral finasteride formulation for the treatment of male AGA patients. To the best of our knowledge, this is the first study that compared these formulations. The results demonstrated that dutasteride 0.05% topical solution was more effective than oral finasteride (1 mg/day) in treating AGA patients.

In the present study, all active treatment groups, dutasteride topical solutions at concentrations of 0.01%, 0.02%, and 0.05%, as well as finasteride, demonstrated significant improvement in TAHC within 1 cm² at the vertex by week 24 compared to baseline, corroborating the established efficacy of 5ARIs in treating AGA. Notably, the dutasteride 0.05% topical solution group exhibited the most substantial improvement in TAHC at week 24, significantly outperforming placebo, finasteride, and other dutasteride groups. Our findings of dutasteride 0.05% topical solution align with a study by Olsen et al., which showed that 2.5 mg doses of oral dutasteride significantly improved hair count compared to 5 mg finasteride at 12 weeks and 24 weeks [16]. Harcha et al. found that orally administered 0.5 mg dutasteride significantly increased hair count and width in a 2.54-cm diameter at week 24 than finasteride (1 mg/d) and placebo [14]. Similarly, Jung et al. reported that treating 31 Korean men with AGA, who had not responded to oral finasteride 1 mg in six months, treatment with 0.5 mg/day dutasteride led to a 10.3% increase in hair density and an 18.9% increase in hair thickness after six months [24]. These studies collectively demonstrate the efficacy of dutasteride over finasteride in enhancing hair growth in patients with AGA. Of interest is the comparison of data between topical dutasteride and topical finasteride. The change from baseline in TAHC at week 24 in our study with topical dutasteride at all tested strengths (0.01%, 0.02%, and 0.05%) was higher than previously reported data of topical finasteride spray solution (1-4 sprays, or 50-200 μL of solution depending on the extent of hair loss) (32.32%, 27.48%, and 75.52% vs 20.20%) [21].

Hair width is another critical factor in the perceived density and overall appearance of hair. The present study revealed that only the dutasteride 0.05% topical solution group showed a significant increase in TAHW compared to placebo at week 24. This improvement in hair width was not significantly different among the various concentrations of dutasteride, suggesting that higher concentrations of dutasteride may offer additional benefits beyond increasing hair count. Moreover, dutasteride 0.05% topical solution and finasteride showed similar efficacy in improving TAHW. In contrast to our findings, a previous study demonstrated that dutasteride 0.5 mg significantly increased hair width in a 2.54-cm diameter at week 24 than finasteride [14]. Another striking finding of the present study is that the dose-dependent increase in hair count and width with topical dutasteride (0.05%) compared to placebo at week 24. This aligns with earlier studies on oral dutasteride conducted by Olsen et al. (at 0.05, 0.1, 0.5, and 2.5 mg dose levels) and Harcha et al. (at 0.02, 0.1, or 0.5 mg/day dose levels) [14,16]. The adjusted mean change from baseline to week 24 in TAHW indicated negligible changes with topical finasteride spray solution in a previous study [21].

In the MHGQ score assessment, the highest proportion of patients was satisfied with dutasteride 0.05% topical solution as compared to finasteride at both week 12 and week 24, supporting objective efficacy data. This alignment between subjective satisfaction and objective outcomes underscores the perceived benefits of dutasteride 0.05% topical solution. Furthermore, the objective assessment of a GPA score of ≥+2 was predominantly achieved in patients using dutasteride 0.05% topical solution at the same time points, reinforcing its effectiveness in improving hair growth. Similar subjective and objective improvement in hair growth were reported with dutasteride compared to finasteride in previously published studies [14,16].

Overall, topical dutasteride and oral finasteride were well-tolerated, with minimal dermal irritation and no significant adverse events or withdrawals, thereby confirming the favourable safety profile of both topical dutasteride and oral finasteride. On the other side, treatment-related sexual adverse events (sexual dysfunction, erectile dysfunction, decreased libido, loss of libido) were reported in 2.8% patients treated with topical finasteride spray solution [21] and 10.81% patients treated with 1 mg oral finasteride daily [15].

Lowering scalp DHT levels is critical for treating AGA due to DHT's role in inhibiting Wnt/β-catenin expression and affecting notch signalling, leading to hair follicle miniaturization [25]. Oral dutasteride at a dose of 0.5 mg/day has been shown to reduce serum DHT levels by more than 90%, compared to a 71.4% reduction with oral finasteride at a dose of 1 mg/day [26]. In the present study, however, no significant changes in either serum testosterone or DHT concentrations were observed with dutasteride-treated groups up to week 12 and week 24, whereas a modest increase in testosterone and a modest reduction in DHT were observed with the reference finasteride-treated group. These modest changes in testosterone and DHT observed with 0.05% topical dutasteride solution could be attributed to the less systemic exposures and thereby less possibility of systemic side effects, which fulfils the objective of the current research investigation. The promising efficacy of 0.05% dutasteride topical solution shown herein supports the dual role of type I and type II 5 alpha-reductase in the pathogenesis of male AGA. We also conducted pharmacokinetic analyses, and the observed plasma profile with concentrations of 0.05% topical dutasteride maintained over 24 hours supports the proposed once-daily dosing regimen. Moreover, detectable plasma levels at day 168 indicate prolonged systemic exposure and support the long-lasting effect of the drug. Overall, the pharmacokinetic data were satisfactory over the first 24-hour period and up to day 168.

The present study also has certain limitations. The study population lacked broad ethnic and gender diversity as the subjects were of Indian origin, and the follow‑up period was relatively short (following 24 weeks of treatment). However, the authors would like to confirm that the placebo arm was not underpowered relative to the active treatment groups in a phase II study; moreover, it is unethical to expose a larger number of placebo arm subjects to no treatment for such a six-month study duration. Improvement in hair loss treatment was not assessed by trichoscopy, a diagnostic tool that reflects the pathophysiology of AGA and is widely used in its diagnosis. In addition, the long-term efficacy and safety of dutasteride in comparison with finasteride are yet to be studied. Further head-to-head comparisons and high-quality randomized controlled trials with larger cohorts are warranted to substantiate the effectiveness and safety profiles of dutasteride and finasteride in treating AGA.

## Conclusions

In male AGA patients, dutasteride 0.05% topical solution (0.5 mg/day) has demonstrated greater efficacy than oral finasteride (1 mg/day). Novel dutasteride topical solution showed a favorable safety and tolerability profile, with no significant adverse effects or skin irritation, which could be attributed to the low systemic exposures. Clinically, there is no standardized dosage for dutasteride; however, some physicians have effectively used oral formulations of dutasteride (0.5 mg/day) for AGA treatment off-label. No specific dosage recommendations for topical dutasteride have been provided in the literature. From our current study findings, it is concluded that further clinical studies are necessary to elucidate the impact of topical dutasteride (0.05%) on AGA treatment outcomes in large-scale Phase III clinical trials.

## Figures and Tables

**Figure 4:**
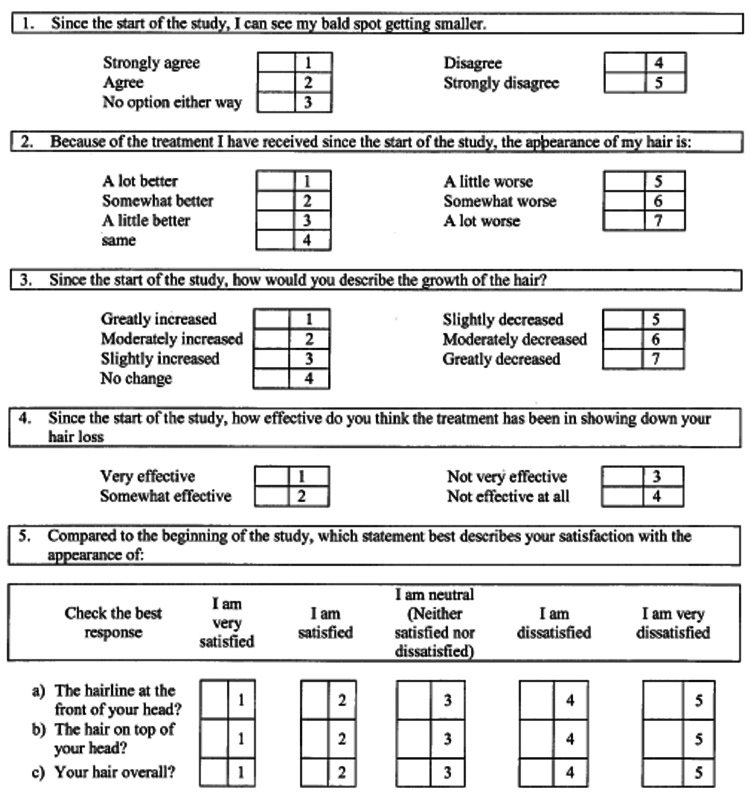
Male hair growth questionnaire used in the study
